# Supplementary material for: The interactome of intact mitochondria by cross-linking mass spectrometry provides evidence for coexisting respiratory supercomplexes
Source: Mol Cell Proteomics. 2017 Dec 8;17(2):216–32. doi: 10.1074/mcp.RA117.000470 (PMC5795388; doi:10.1074/mcp.RA117.000470)
Supplement: Supplemental Data [file supp_RA117.000470_133922_0_supp_23978_fzffwf.zip › spectra_annotation/mito_DR_spectra_annotation/285-1-8-1-10-1.pdf]

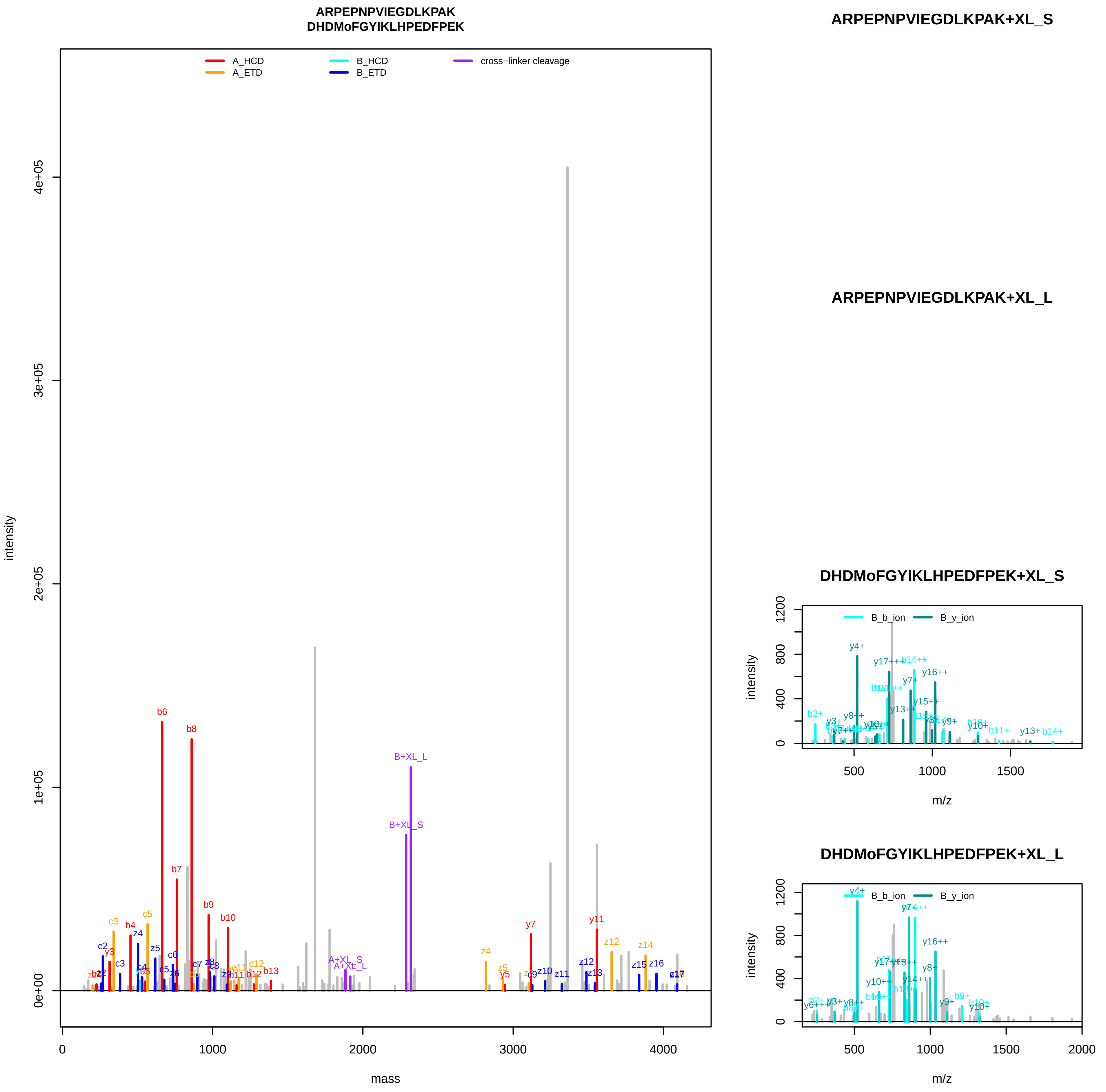
ARPEPNPVIEGDLKPAK
DHDMoFGYIKLHPEDFPEK
A_HCD
A_ETD
B_HCD
B_ETD
cross−linker cleavage
intensity
0e+00
1e+05
2e+05
3e+05
4e+05
mass
0
1000
2000
3000
4000
b6
b8
b7
b9
b10
b4
c3
c5
z4
c2
y3
z5
c6
c3
c7
c8
z8
b12
b13
c12
B+XL_L
B+XL_S
A+XL_S
A+XL_L
y7
y11
z4
z5
y5
z12
z14
z10
z11
z12
z13
z15
z16
z17
ARPEPNPVIEGDLKPAK+XL_S
ARPEPNPVIEGDLKPAK+XL_L
DHDMoFGYIKLHPEDFPEK+XL_S
B_b_ion
B_y_ion
intensity
0
400
800
1200
m/z
500
1000
1500
y4+
y17+++
b14++
y16++
y7+
y15++
y13++
b2+
y8++
y3+
y9+
b10+
y10+
b11+
y13+
b14+
DHDMoFGYIKLHPEDFPEK+XL_L
B_b_ion
B_y_ion
intensity
0
400
800
1200
m/z
500
1000
1500
2000
y4+
y7+++
y16++
y17++
y18++
y8+
y10++
y14++
y3+
y8++
y9+
b9+
b10+
y10+
y6+++
